# Clinical and echocardiographic changes after intermittent levosimendan infusion in patients with advanced heart failure

**DOI:** 10.1093/eschf/xvag017

**Published:** 2026-01-13

**Authors:** Mauro Riccardi, Emilia D’Elia, Fabio Fazzari, Camilla Cirelli, Stefano Bisegna, Maria Giulia Bellicini, Matteo Pagnesi, Savina Nodari, Daniela Pini, Renato M Bragato, Michele Senni, Carlo M Lombardi, Marco Metra, Riccardo M Inciardi

**Affiliations:** Department of Medical and Surgical Specialties, Radiological Sciences and Public Health, Institute of Cardiology, University of Brescia, Spedali Civili di Brescia, Brescia, Italy; Division of Cardiology, Cremona Hospital, Largo Priori Emilio, 1, 26100 Cremona, Italy; Department of Cardiology, Papa Giovanni XXIII Hospital, Bergamo, Italy; Perioperative Cardiology and Cardiovascular Imaging Department, Centro Cardiologico Monzino IRCCS, Milan, Italy; Department of Cardiology, Papa Giovanni XXIII Hospital, Bergamo, Italy; Department of Medical and Surgical Specialties, Radiological Sciences and Public Health, Institute of Cardiology, University of Brescia, Spedali Civili di Brescia, Brescia, Italy; Cardiologia, Ospedale Maggiore, Lodi, Italy; Department of Medical and Surgical Specialties, Radiological Sciences and Public Health, Institute of Cardiology, University of Brescia, Spedali Civili di Brescia, Brescia, Italy; Department of Medical and Surgical Specialties, Radiological Sciences and Public Health, Institute of Cardiology, University of Brescia, Spedali Civili di Brescia, Brescia, Italy; Department of Medical and Surgical Specialties, Radiological Sciences and Public Health, Institute of Cardiology, University of Brescia, Spedali Civili di Brescia, Brescia, Italy; Cardiology Unit, Fondazione IRCCS San Gerardo dei Tintori, Monza, Italy; Humanitas Research Hospital IRCCS, Rozzano, Milan, Italy; Department of Cardiology, Papa Giovanni XXIII Hospital, Bergamo, Italy; Department of Cardiovascular Medicine, University Bicocca Milan, Milan, Italy; Department of Medical and Surgical Specialties, Radiological Sciences and Public Health, Institute of Cardiology, University of Brescia, Spedali Civili di Brescia, Brescia, Italy; Division of Cardiology, Cremona Hospital, Largo Priori Emilio, 1, 26100 Cremona, Italy; Department of Medical and Surgical Specialties, Radiological Sciences and Public Health, Institute of Cardiology, University of Brescia, Spedali Civili di Brescia, Brescia, Italy; Department of Medical and Surgical Specialties, Radiological Sciences and Public Health, Institute of Cardiology, University of Brescia, Spedali Civili di Brescia, Brescia, Italy

**Keywords:** Levosimendan, AdHF, Echocardiography, GLS, FWLS

## Abstract

**Introduction:**

To evaluate the short-term clinical, structural, and functional cardiac changes following intermittent levosimendan infusion and their prognostic implications in patients with advanced heart failure (AdHF).

**Methods:**

In this prospective, multicentre, observational study, patients with AdHF treated with intermittent levosimendan infusion (12.5 mg at a rate of 0.1 μg/kg/min, without a bolus) were enrolled. Clinical, laboratory, and comprehensive echocardiographic assessments were performed at baseline, 48 h, and 30 days post-infusion. The primary endpoint was to evaluate the extent of clinical and cardiac structure and function changes during the first 30 days after levosimendan infusion. Secondary outcome of interest was the association of cardiac changes and a composite endpoint of decompensated HF, urgent HF rehospitalization, cardiogenic shock, cardiac arrest, and cardiovascular death at 6 months.

**Results:**

A total of 37 patients with AdHF were included (mean age 71.1 ± 9.0 years; 59.5% male). A non-significant trend reduction in mean arterial pressure values was observed over time (81.3 ± 9.5 mmHg vs 80.9 ± 7.7 mmHg after 48 h vs 79.1 ± 6.9 mmHg at 30 days, *P*-value for trend = .079). NT-proBNP levels significantly decreased at 48 h and rose again by 30 days [from 1802 (1127–4436) ng/l to 1060 (830–3864) ng/l at 48 h and 1661 (1015–4245) ng/l at 30 days, *P*-value for trend = .006]. The main left ventricular (LV) and right ventricular (RV) structure and function parameters improved at 48 h but returned to baseline or a slight improvement by 30 days (LV ejection fraction: 30.9 ± 11.3% vs 32.2 ± 11.2%, vs 30.9 ± 11.3%, *P*-value for trend = .001; LV global longitudinal strain: −8.0 ± 3.5% vs −9.2 ± 3.5% vs −8.0 ± 3.3%, *P*-value for trend < .001; tricuspid annular plane systolic excursion: 16.9 ± 3.1 mm vs 17.7 ± 3.4 vs 17.0 ± 3.5, *P*-value for trend .007; systolic pulmonary artery pressure: 48.7 ± 15.8 mmHg vs 41.6 ± 12.7 mmHg vs 46.1 ± 15.0, *P*-value for trend <.001). Overall, 17 (45.9%) patients experienced CV events. After accounting for confounders, ΔLV global longitudinal strain (GLS) after 48 h (adjusted odds ratio (OR) 0.26, 95% confidence interval (CI) 0.10–0.67, *P* = .006) and ΔRV free wall longitudinal strain (FWLS) (adjusted OR 0.75, 95% CI 0.57–0.99, *P* = .041) were significantly associated with a lower risk of events.

**Conclusions:**

In patients with AdHF, intermittent levosimendan infusion resulted in early improvements in NT-proBNP levels and biventricular function, which tended to wane by 30 days. Early enhancements in LVGLS and RVFWLS were independently associated with better clinical outcomes, suggesting their potential role as markers to guide patient selection and therapeutic response.

## Introduction

Advanced heart failure (HF) (AdHF) represents an end-stage phase of HF characterized by persistent symptoms with severe impairment of exercise capacity and multiple unplanned visits or hospitalizations due to congestion, low cardiac output or severe arrhythmias.^1^ The overall prevalence of AdHF is expected to increase due to the HF burden and the efficacy of available guideline-directed medical therapy (GDMT).^1,2^ Yet, prognosis remains poor with a 1-year mortality ranging from 25% to 75%.^1^ Although disease-modifying medical therapies represents the cornerstone of AdHF treatment, these patients may require vasoactive medication or short-term mechanical circulatory support (MCS) to stabilize clinical condition and end-organ function while awaiting long-term MCS implantation or heart transplantation (HTx).^1,2^ Vasoactive medications may also be used as a bridge therapy or palliative support in patients not eligible for HTx or long-term MCS. Levosimendan, a calcium sensitizer that can increase cardiac inotropy through a direct effect on cardiac troponin-C,^3^ has been tested in patients with AdHF in several trials.^4–7^ Despite levosimendan’s short half-life of 1 h, its effect can last for weeks due to the longer (70–90 h) half-life of its active metabolite (OR-1896).^8^ Overall, intermittent infusion of levosimendan has been shown mainly an improvement in symptoms and quality of life.^6,9–11^ Few studies investigated the changes of clinical and cardiac structure and function parameters in AdHF patients treated with intermittent infusions of levosimendan.^12–14^

We therefore investigated the effect of levosimendan infusion on cardiac remodelling and its prognostic implication in a population with AdHF.

## Methods

This is a prospective, multicentre, observational study conducted in three tertiary Italian Centre (IRCCS Humanitas Research Hospital, Rozzano-Milan, Italy; ASST Spedali Civili, Brescia, Italy; and ASST Papa Giovanni XXIII, Bergamo, Italy), enrolling patients with AdHF who had to start intermittent levosimendan infusion between June 2022 and June 2023. Advanced heart failure was defined according to 2021 European Society of Cardiology Guideline for HF treatment.^1^ Patients with acute decompensated HF or cardiogenic shock (CS) treated with levosimendan in an acute setting were excluded, as well as patients with poor acoustic windows and patients in which GDMT has been titrated. The main purpose of levosimendan treatment was palliative, as no patient was eventually treated as bridge to HTx or MCS. Clinical and laboratory data were collected at the time of the admission, after 48 h and after 1 month. At the same day of admission, every patient received 12.5 mg of levosimendan infusion at a rate of 0.1 μg/kg/min, without a bolus. A complete clinical and echocardiographic examination was performed before levosimendan infusion. A clinical and echocardiographic follow-up was performed at 30 days. The study complied with the Declaration of Helsinki and was approved by the local ethics committee.

### Clinical characteristics and laboratory findings

Clinical data including age, gender, cardiovascular risk factors, cardiac history and procedures, medical therapy, INTERMACS class and New York Heart Association (NYHA) functional class were collected at admission. All patients were under maximal tolerated GDMT, in accordance with the current recommendations.^1^ Clinical status was checked and recorded at each planned visit in terms of NYHA class and body weight variations. Vital parameters including blood pressure and heart rate were recorded too. Laboratory data were obtained from each patient at the time of admission, 48 h and 30 days including N-terminal pro b-type natriuretic peptide (NT-proBNP), troponin, and creatinine.

### Echocardiographic analysis

At admission at 48 h and at 1 month after levosimendan infusion, all patients underwent comprehensive transthoracic echocardiography, under rest conditions in the left lateral decubitus position, using a commercially available system (Vivid E95; GE Healthcare, Chicago, IL, USA; Epiq Affiniti 7C; Philips Healthcare, Amsterdam, Netherlands) equipped with a 3.5 MHz multiphase array probe. All measurements were performed by experienced operators. Electrocardiogram-triggered echocardiographic data were digitally stored on a dedicated workstation for offline analysis with EchoPAC software Version 203 (GE Healthcare, Horten, Norway) and IntelliSpace Cardiovascular version 5 (Philips Healthcare, Amsterdam, Netherlands). All recordings and measurements were performed according to current guidelines.^15^ Cardiac dimensions (both for right and left ventricles and atria) and function were measured from apical views and left ventricular (LV) ejection fraction (LVEF) was calculated using the Simpson biplane method. Peak early (E) and late diastolic flow velocity (A) were measured from the apical 4-chamber view by pulsed-wave Doppler. Tissue Doppler imaging was used to assess peak early diastolic tissue velocity (*e*′) at the lateral and septal mitral annulus. The average *E*/*e*′ ratio was calculated to estimate LV filling pressure according to recommendations. In patients with detectable tricuspid regurgitation, systolic pulmonary artery pressure (sPAP) was estimated using the maximal velocity of the tricuspid regurgitation jet and an estimation of the right atrial pressure dictated by the size and collapsibility of the inferior vena cava. The operators, during the execution of echocardiography, were blinded with respect to previous echocardiographic values, laboratory analysis and the patient’s clinical state.

### Strain analysis

During the examination, two-dimensional grey-scale images were acquired in the apical 4-, 3-, and 2-chamber views at a framerate of 70–90 fps. These recordings were processed offline using a dedicated software (EchoPAC version 203, GE Healthcare, Horten, Norway; IntelliSpace Cardiovascular version 5, Philips Healthcare, Amsterdam, Netherlands) to calculate LV global longitudinal strain (LVGLS), right ventricular (RV) free wall longitudinal strain (RVFWLS), and RV septal longitudinal strain (RVSLS). Left ventricular global longitudinal strain was calculated as the average of the peak systolic longitudinal strain of all segments. Right ventricular free wall longitudinal strain and RVSLS were calculated through a dedicated view of the right ventricle.

### Study endpoint

The primary endpoint was to evaluate the extent of cardiac remodelling during the first 30 days after levosimendan infusion. We also explored the association of cardiac remodelling, defined as an improvement in echocardiographic parameters compared with baseline, with clinical outcomes including a composite endpoint of decompensated HF, urgent HF rehospitalization, CS, cardiac arrest, and cardiovascular death up to 6 months from enrolment. Decompensated HF was defined as a worsening of the HF status and/or NYHA functional class with clinical evidence of peripheral and/or central congestion needing intravenous diuretic therapy or an increase of oral diuretic therapy during unplanned ambulatory visit. Urgent HF rehospitalization was defined as an unplanned readmission to the hospital due to an acute HF decompensation. Cardiogenic shock was defined by the presence of systolic blood pressure <90 mmHg for more than 30 min or vasopressor support to maintain systolic blood pressure >90 mmHg along with evidence of end-organ damage. Cardiac death was defined as death due to cardiovascular causes (myocardial infarction, sudden cardiac death, CS, and pulmonary oedema).

### Statistical analysis

Continuous variables are expressed as mean ± standard deviation (SD) or median and interquartile range (IQR), as appropriate, and were compared with the Student T-test or Mann–Whitney *U* test, respectively. Categorical variables are presented as numbers and percentages and were compared with the χ^2^ or Fisher test, as appropriate. The significance of the variations in clinical and echocardiographic parameters in the three-time measurements was evaluated with multivariate mean equality test. Clinical and echocardiographic data at admission and variations between echocardiographic parameters post- and pre-levosimendan infusion (Δvariable) were compared between patients with composite outcome and those without composite outcome. Moreover, the association between composite outcome and the variations in selected echocardiographic parameters (LVEF, LVGLS, systolic pulmonary artery pressure, LV outflow tract—velocity time integral, LV end-diastolic volume [LVEDV] indexed, and left atrial volume indexed) between admission, 48 h and 30 days, was analysed with repeated measures mixed models. Similarly, the association between NT-proBNP levels and the same echocardiographic parameters between admission, 48 h and 30 days, was analysed with repeated measures mixed models. Univariable and multivariable binary logistic regression analyses were performed to evaluate the impact of Δvariable and composite outcome. Age and sex were included in the multivariable analyses. Results of the analyses are reported as unadjusted or adjusted odds ratio (OR) with 95% confidence interval (CI). A logistic spline curve was created to visualize the probability of change in echocardiographic parameters and CV outcomes. The number of knots that provided the lowest AIC and showed statistical significance was chosen.

Analyses were performed with STATA software version 18 (STATA Corp., College Station, Texas, USA). Statistical significance was set at the two-tailed 0.05 level.

## Results

### Demographic and clinical characteristics

A total of 37 patients with AdHF were included in the present study. Overall, mean age was 71.1 ± 9.0 years and 59.5% of patients were males. Mean INTERMACS was 3 ± 1 and 62.2% had NYHA Class III. Baseline characteristics, medical therapy and laboratory tests are reported in *Table 1*. Non-ischaemic cardiomyopathy was the cause of AdHF in 27% of patients, coronary artery disease in 62.2% and a primary valvular heart disease in 10.8%. Mean arterial pressure (MAP) at admission was 81.3 ± 9.5 mmHg, median NT-proBNP values was 1802 ng/l (IQR 1127–4436) and mean estimated glomerular filtration rate calculated with CKD-EPI was 42.1 ± 20.8 ml/min. Regarding medical therapy at baseline (*Table 1*), 54.1% of patients were treated with sacubitril/valsartan, 94.6% with mineralocorticoid receptor antagonists, 91.9% with β-blockers and 56.8% with sodium-glucose cotransporter-2 inhibitors. Overall, 17 (45.9%) patients experienced the composite endpoint. Main clinical characteristics according to outcomes are reported in Supplementary Table S1.

**Table 1:** Demographic and clinical characteristics of the overall population

Demographics and clinical characteristics	
Age, years	71.1 ± 9.0
Male, *n* (%)	22 (59.5)
BMI, Kg/m^2^	25.9 ± 4.8
NYHA Class III	23 (62.2)
INTERMACS	3 ± 1
SBP, mmHg	111.6 ± 14.1
MAP, mmHg	81.3 ± 9.5
Heart rate, bpm	67.7 ± 10.9
Hypertension, *n* (%)	17 (46.0)
Dyslipidaemia, *n* (%)	21 (56.8)
Diabetes, *n* (%)	20 (54.1)
Coronary artery disease, *n* (%)	23 (62.2)
Prior STEMI, *n* (%)	15 (40.5)
Atrial fibrillation, *n* (%)	23 (62.2)
Chronic kidney disease, *n* (%)	31 (83.8)
Stage III	23 (62.2)
Stage IV	7 (18.9)
COPD, *n* (%)	11 (29.7)
Chronic anaemia, *n* (%)	6 (16.2)
Non-ischaemic cardiomyopathy, *n* (%)	10 (27)
Primary VHD, *n* (%)	4 (10.8)
ICD, *n* (%)	18 (48.7)
CRT-D, *n* (%)	17 (46)
MitraClip, *n* (%)	3 (8.1)
Medical therapy	
ACEi, *n* (%)	1 (2.7)
ARB, *n* (%)	2 (5.4)
ARNI, *n* (%)	20 (54.1)
MRA, *n* (%)	35 (94.6)
β-Blockers, *n* (%)	34 (91.9)
SGLT2i, *n* (%)	21 (56.8)
Diuretics, *n* (%)	37 (100)
Ivabradine, *n* (%)	3 (8.1)
Laboratory tests	
NT-proBNP, ng/l	1802 (1127–4436)
Hs-TnT, ng/l	20 (14–38)
Creatinine, mg/dl	1.87 ± 0.83
eGFR, ml/min	42.1 ± 20.8

Data are presented as *n* (% on available), as mean (±SD) and as median (IQR).

ACEi, angiotensin converting enzyme inhibitor; ARB, angiotensin receptor blocker; ARNI, angiotensin receptor neprilysin inhibitor; BMI, body mass index; COPD, chronic obstructive pulmonary disease; CRT-D, cardiac resynchronization therapy—defibrillator; eGFR, estimated glomerular filtration rate; hs-TnT, high-sensitivity cardiac troponin T; ICD, implantable cardioverter defibrillator; MAP, mean arterial pressure; MRA, mineralocorticoid receptor antagonist; NYHA, New York Heart Association; NT-proBNP, N-terminal pro b-type natriuretic peptide; SBP, systolic blood pressure; SGLT2i, sodium-glucose cotransporter 2 inhibitor; STEMI, ST elevation myocardial infarction; VHD, valvular heart disease.

Echocardiographic parameters at baseline are reported in *Table 2*. Mean LVEF was 30.9 ± 11.3% with a mean LVEDV indexed of 107.2 ± 40.3 ml/m^2^. Mean left atrial volume indexed was 61.0 ± 18.9 ml/m^2^. 12 patients (32.4%) of patients had moderate-severe mitral regurgitation. Regarding RV function, mean FAC was 34.4 ± 10.7% and RVFWLS was −17.7 ± 6.7%. Mean sPAP was 48.7 ± 15.8 mmHg. Echocardiographic parameters according to outcomes are reported in Supplementary Table S2.

**Table 2:** Baseline echocardiographic parameters

	Overall population (*n* = 37)
LVEDV, ml	194.8 ± 75.8
LVEDVi, ml/m^2^	107.2 ± 40.3
LV-EDD, mm	62.2 ± 9.5
LVEF, %	30.9 ± 11.3
Stroke volume, ml	14.8 ± 4.7
LVOT-VTI, cm	50.4 ± 14.4
LVGLS, %	−8.0 ± 3.5
LAV, ml	110.9 ± 38.0
LAVi ml/m^2^	61.0 ± 18.9
RAV, ml	80.7 ± 58.7
*E*/*e*′	17.6 ± 7.4
Moderate-severe MR, *n* (%)	12 (32.4)
TAPSE, mm	16.9 ± 3.1
S′wave TDI, cm/s	8.9 ± 2.0
FAC, %	34.4 ± 10.7
sPAP, mmHg	48.7 ± 15.8
TAPSE/sPAP, mm/mmHg	0.38 ± 0.15
RVFWLS, %	−17.7 ± 6.7
RVSLS, %	8.5 ± 5.1

Data are presented as *n* (% on available) and as mean (±SD).

EDD, end-diastolic diameter; EDV, end-diastolic volume; EDVi, end-diastolic volume indexed; FAC, fractional area change; FWLS, free wall longitudinal strain; GLS, global longitudinal strain; LAV, left atrium volume; LAVi, left atrium volume indexed; LV, left ventricle; LVEF, left ventricular ejection fraction; LVOT-VTI, left ventricular outflow tract—velocity time integral; MR, mitral regurgitation; RAV, right atrium volume; RV, right ventricle; SLS, septal longitudinal strain; sPAP, systolic pulmonary artery pressure; TAPSE, tricuspid annulus plane systolic excursion; TDI, tissue Doppler imaging.

### Clinical, laboratory and echocardiographic variations during the 30 days

**Figure 1:**
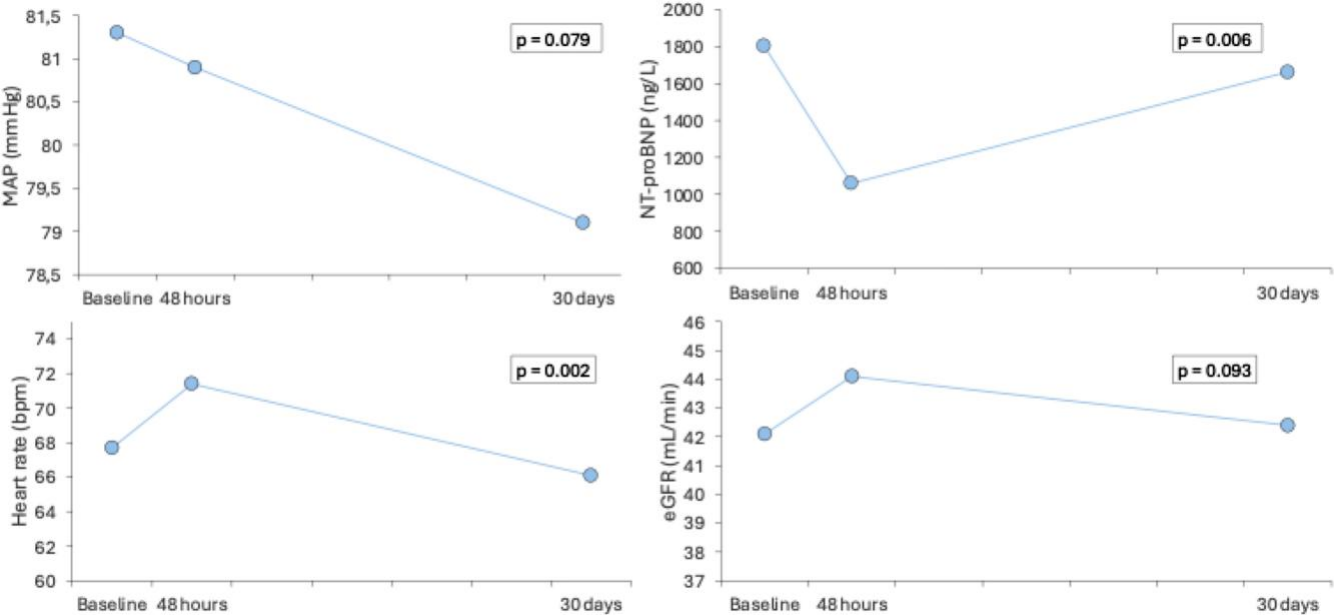
Clinical and laboratory variations during the 30 days after levosimendan infusion. eGFR, estimated glomerular filtration rate; MAP, mean arterial pressure

**Figure 2:**
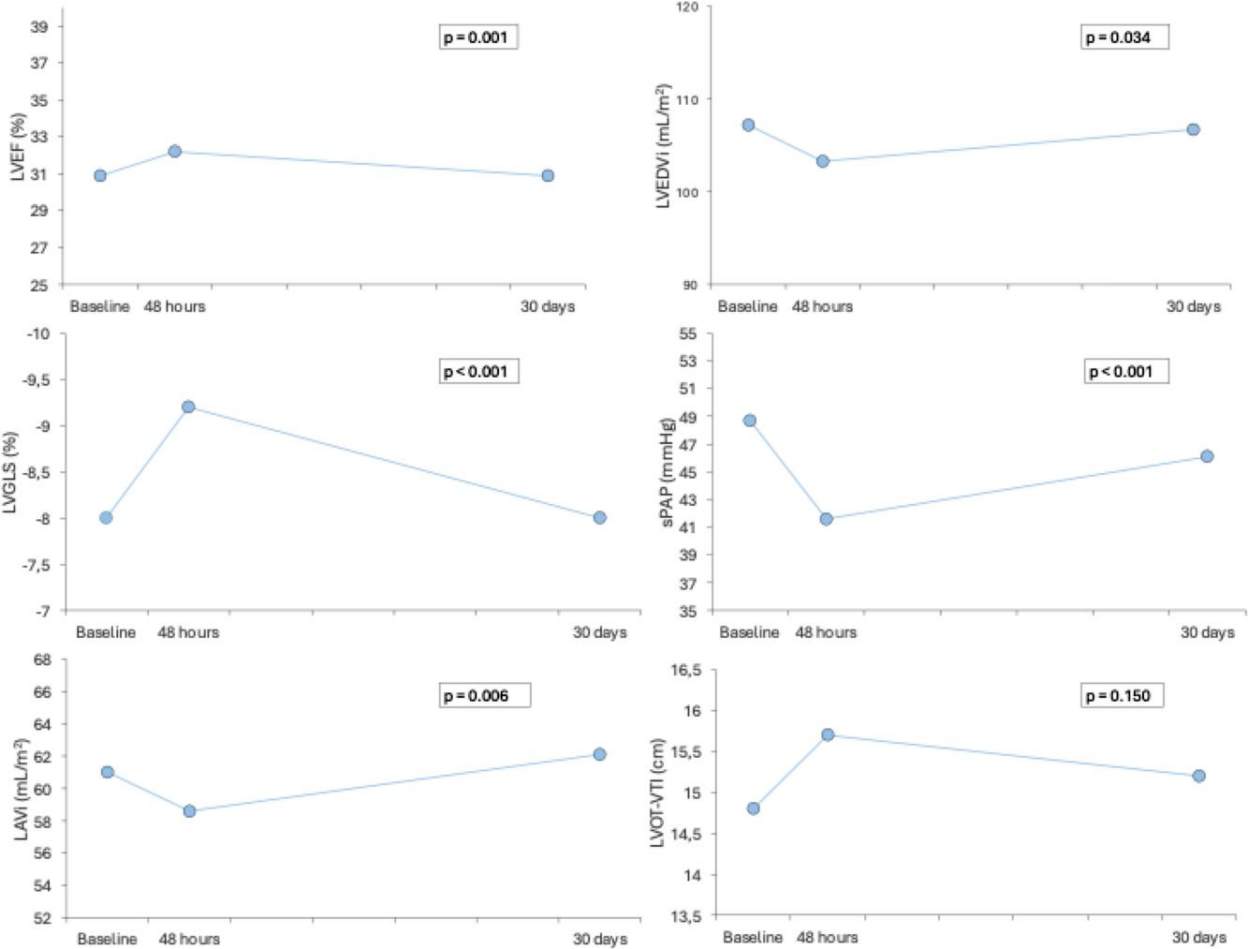
Echocardiographic variations during the 30 days after levosimendan infusion. LAVi, left atrial volume indexed; LVEDVi, left ventricular end-diastolic volume indexed; LVEF, left ventricular ejection fraction; LVGLS, left ventricular global longitudinal strain; LVOT-VTI, left ventricular outflow tract—velocity time integral; sPAP, systolic pulmonary arterial pressure

Clinical, laboratory and echocardiographic variations during the 30 days after levosimendan infusion are reported in *Tables 3–4* and *Figures 1–2*. Overall, patients showed a non-significant reduction in MAP values at 30 days (81.3 ± 9.5 mmHg vs 80.9 ± 7.7 mmHg vs 79.1 ± 6.9 mmHg, *P*-value for trend = .079). Heart rate initially increased at 48 h after levosimendan infusion with a progressive reduction as observed at baseline (67.7 ± 10.9 bpm vs 71.4 ± 10.7 bpm vs 66.1 ± 9.1 bpm, *P*-value for trend = .002). Similarly, NT-proBNP was significantly reduced after 48 h of levosimendan infusion with an increase at 30 days without reaching baseline values [1802 (1127–4436) ng/l vs 1060 (830–3864) ng/l vs 1661 (1015–4245) ng/l, *P*-value for trend = .006]. The estimated glomerular filtration rate increased between baseline and 48-h measurements (42.1 ± 20.8 ml/min vs 44.1 ± 22.3 ml/min, *P*-value .032), with a decline after 30 days as observed at baseline (42.4 ± 21.5 ml/min). No differences were found for systolic blood pressure and high-sensitivity cardiac troponin-C.

**Table 3:** Changes in main clinical and laboratory parameters

	Baseline	48 h	30 days	*P*-value baseline—48 h	*P*-value 48 h—30 days	*P*-value trend
Clinical parameters and laboratory tests						
SBP, mmHg	111.6 ± 14.1	109.2 ± 12.2	110.4 ± 11.2	.089	.518	.233
MAP, mmHg	81.3 ± 9.5	80.9 ± 7.7	79.1 ± 5.5	.733	.185	.079
Heart rate, bpm	67.7 ± 10.9	71.4 ± 10.7	66.1 ± 9.1	**.004**	**<**.**001**	.**002**
NT-proBNP, ng/l	1802 (1127–4436)	1060 (830–3864)	1661 (1015–4245)	.**002**	.**003**	.**006**
Creatinine, mg/dl	1.87 ± 0.83	1.76 ± 0.68	1.81 + 0.65	.149	.209	.287
eGFR, ml/min	42.1 ± 20.8	44.1 ± 22.3	42.4 ± 21.5	.278	.**032**	.093
Hs-TnT, ng/l	20 (14–38)	—	19 (13–40)	—	—	.167

Data are presented as mean (±SD) and as median (IQR). Bold values represent significant *P*-values.

eGFR, estimated glomerular filtration rate; hs-TnT, high-sensitivity cardiac troponin T; MAP, mean arterial pressure; NT-proBNP, N-terminal pro b-type natriuretic peptide; SBP, systolic blood pressure.

**Table 4:** Changes in cardiac structure and function

	Baseline	48 h	30 days	*P*-value baseline—48 h	*P*-value 48 h—30 days	*P*-value trend
LVEDV, ml	194.8 ± 75.8	187.6 ± 73.9	194.0 + 74.6	**.008**	.**034**	.**024**
LVEDVi, ml/m^2^	107.2 ± 40.3	103.3 ± 39.1	106.7 ± 39.4	.**012**	.**047**	.**034**
LV-EDD, mm	62.2 ± 9.5	61.5 ± 9.8	62.2 ± 9.6	.**010**	.052	.**026**
LVEF, %	30.9 ± 11.3	32.2 ± 11.2	30.9 ± 11.3	.**004**	**<**.**001**	.**001**
Stroke volume, ml	50.4 ± 14.4	51.8 ± 13.0	52.1 ± 14.5	.258	.901	.177
LVOT-VTI, cm	14.8 ± 4.7	15.7 ± 4.6	15.2 ± 4.6	.166	.594	.150
LVGLS, %	−8.0 ± 3.5	−9.2 ± 3.5	−8.0 ± 3.3	**<**.**001**	**<**.**001**	**<**.**001**
LAV, ml	110.9 ± 38.0	106.5 ± 38.7	112.9 ± 37.2	.**005**	.**001**	.**005**
LAVi, ml/m^2^	61.0 ± 18.9	58.6 ± 19.4	62.1 ± 18.4	.**004**	.**002**	.**006**
RAV, ml	80.7 ± 58.7	80.3 ± 56.0	82.7 ± 58.6	.636	.364	.663
*E*/*e*′	17.6 ± 7.4	15.3 ± 7.6	16.3 ± 7.1	.**008**	.296	.**018**
Moderate-severe MR, *n* (%)	11 (29.7)	12 (32.4)	11 (29.7)	.317	.655	.619
TAPSE, mm	16.9 ± 3.1	17.7 ± 3.4	17.0 ± 3.5	.**004**	.**007**	.**007**
S’wave TDI, cm/s	8.9 ± 2.0	9.6 ± 2.4	9.2 ± 2.2	.**001**	.**041**	.**006**
FAC, %	34.4 ± 10.7	36.0 ± 11.8	34.9 ± 10.9	.**035**	.128	.056
sPAP, mmHg	48.7 ± 15.8	41.6 ± 12.7	46.1 ± 15.0	**<**.**001**	.**016**	**<**.**001**
TAPSE/sPAP, mm/mmHg	0.38 ± 0.15	0.46 ± 0.18	0.40 ± 0.15	**<**.**001**	.**006**	.**001**
RVFWLS, %	−17.7 ± 6.7	−19.2 ± 7.3	−18.2 ± 7.0	.**022**	.180	.115
RVSLS, %	8.5 ± 5.1	9.6 ± 5.0	9.4 ± 5.0	.**036**	.844	.093

Data are presented as *n* (% on available) and as mean (±SD). Bold values represent significant *P*-values.

EDD, end-diastolic diameter; EDV, end-diastolic volume; EDVi, end-diastolic volume indexed; FAC, fractional area change; FWLS, free wall longitudinal strain; GLS, global longitudinal strain; LAV, left atrium volume; LAVi, left atrium volume indexed; LV, left ventricle; LVEF, left ventricular ejection fraction; LVOT-VTI, left ventricular outflow tract—velocity time integral; MR, mitral regurgitation; RAV, right atrium volume; RV, right ventricle; SLS, septal longitudinal strain; sPAP, systolic pulmonary artery pressure; TAPSE, tricuspid annulus plane systolic excursion; TDI, tissue Doppler imaging.

The main LV and RV structure and function parameters improved in the first 48 h after levosimendan infusion, but subsequently declined by 30 days, approaching baseline values. Left ventricular ejection fraction increased at 48 h (30.9 ± 11.3% vs 32.2 ± 11.2%, *P* = .004) and then declined to baseline values at 30 days (30.9 + 11.3%, *P* trend = .001). Similar trend also was observed for LVGLS (−8.0 ± 3.5% vs −9.2 ± 3.5% vs −8.0 ± 3.3%, *P*-value for trend <.001), tricuspid annular plane systolic excursion (TAPSE) (16.9 ±3.1 mm vs 17.7 ± 3.4 vs 17.0 ± 3.5, *P*-value for trend .007), sPAP (48.7 ± 15.8 mmHg vs 41.6 ± 12.7 mmHg vs 46.1 ± 15.0, *P*-value for trend <.001), and LVEDV (107.2 ± 40.3 ml/m^2^ vs 103.3 ± 39.1 ml/m^2^ vs 106.7 ± 39.4 ml/m^2^, *P*-value for trend .034).

**Figure 3:**
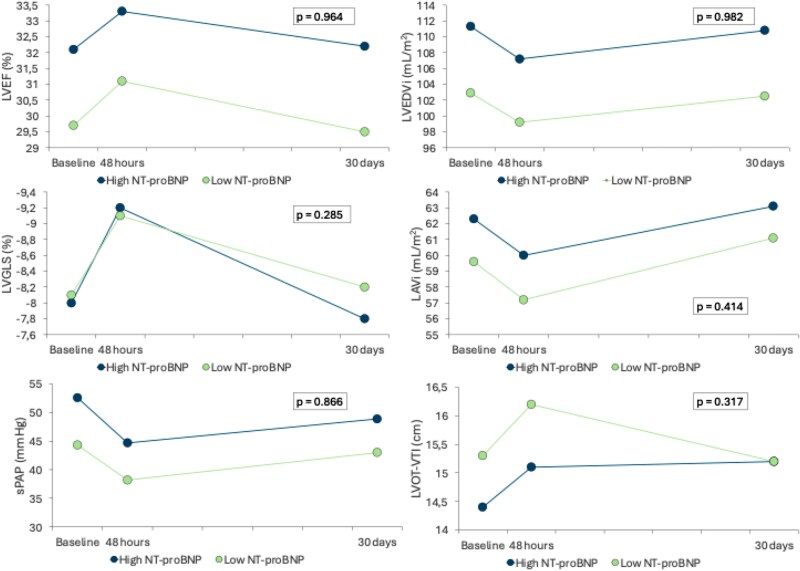
Change in main cardiac parameters was found according to baseline NT-proBNP. LAVi, left atrial volume indexed; LVEDVi, left ventricular end-diastolic volume indexed; LVEF, left ventricular ejection fraction; LVGLS, left ventricular global longitudinal strain; LVOT-VTI, left ventricular outflow tract—velocity time integral; sPAP, systolic pulmonary arterial pressure

No significant change in main cardiac parameters was found according to baseline NT-proBNP (*Figure 3*).

### Association of cardiac changes and CV outcomes

Supplementary Table S3 and Supplementary Fig. S1 reported outcomes according to cardiac changes after 48 h. LVGLS and RVFWLS improvement was higher after levosimendan infusion in patients without CV events as compared with those who experienced CV events (Δ1.61 ± 0.96 vs Δ0.58 ± 1.01, *P* = .003 and Δ2.76 ± 3.47 vs Δ-0.02 ± 3.59, *P* = .022, respectively).

**Figure 4:**
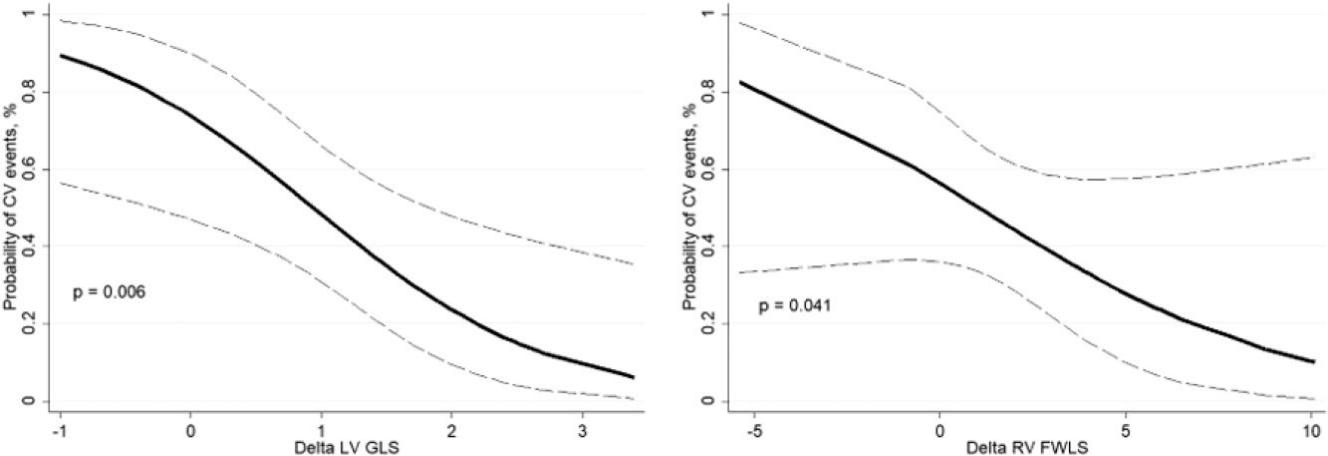
Rates of composite outcome according to LV and RV function changes after 48 h infusion. CV, cardiovascular; LVGLS, left ventricular global longitudinal strain; RVFWLS, right ventricular. free wall longitudinal strain

Univariable and multivariable analysis showing the association of echocardiographic parameters variation post- and pre- levosimendan infusion on composite outcome are reported in *Table 5*. At multivariable analysis, higher ΔLVGLS (adjusted OR 0.26, 95% CI 0.10–0.67, *P* = .006) and higher ΔRVFWLS (adjusted OR 0.75, 95% CI 0.57–0.99, *P* = .041) were significantly associated with a lower risk of composite outcome, such that an increase of LV and RV function were associated with a reduced risk of events (*Figure 4*).

**Table 5:** Association between echocardiographic parameters changes (baseline-48 h) during levosimendan infusion and CV outcomes

Variable	Univariable analysis		Multivariable analysis	
	Unadjusted OR (95% CI)	*P*-value	Adjusted OR^a^ (95% CI)	*P*-value
ΔLVEDV, ml	0.96 (0.89–1.02)	.183	0.96 (0.89–1.03)	.241
ΔLVEDVi, ml/m^2^	0.93 (0.83–1.04)	.217	0.94 (0.83–1.06)	.279
ΔLV-EDD, mm	0.76 (0.46–1.24)	.272	0.75 (0.45–1.26)	.277
ΔLVEF, %	1.18 (0.87–1.62)	.289	1.18 (0.85–1.63)	.315
ΔStroke volume, mL	0.95 (0.86–1.05)	.351	0.94 (0.85–1.04)	.249
ΔLVOT-VTI, cm	0.88 (0.71–1.09)	.246	0.90 (0.72–1.13)	.372
ΔLVGLS, %	0.33 (0.14–0.77)	**.011**	0.26 (0.10–0.67)	.**006**
ΔLAV, ml	0.92 (0.84–1.02)	.103	0.91 (0.82–1.01)	.093
ΔLAVi, ml/m^2^	0.86 (0.72–1.03)	.092	0.84 (0.69–1.02)	.077
ΔRAV, ml	1.18 (0.98–1.42)	.076	1.19 (0.99–1.42)	.062
Δ*E*/*e*′	0.99 (0.87–1.14)	.944	1.00 (0.87–1.15)	.986
ΔTAPSE, mm	0.82 (0.51–1.30)	.390	0.81 (0.50–1.30)	.375
ΔS′wave TDI, cm/s	0.93 (0.56–1.54)	.770	0.98 (0.58–1.66)	.948
ΔFAC, %	0.98 (0.85–1.14)	.839	1.00 (0.85–1.17)	.978
ΔsPAP, mmHg	0.99 (0.92–1.07)	.811	0.99 (0.92–1.07)	.779
ΔRVFWLS, %	0.76 (0.58–0.99)	.**045**	0.75 (0.57–0.99)	.**041**
ΔRVSLS, %	0.84 (0.58–1.21)	.352	0.77 (0.50–1.20)	.257

Data are presented as OR and 95% CI. Bold values represent significant *P*-values.

EDD, end-diastolic diameter; EDV, end-diastolic volume; EDVi, end-diastolic volume indexed; FAC, fractional area change; FWLS, free wall longitudinal strain; GLS, global longitudinal strain; LAV, left atrium volume; LAVi, left atrium volume indexed; LV, left ventricle; LVEF, left ventricular ejection fraction; LVOT-VTI, left ventricular outflow tract—velocity time integral; RAV, right atrium volume; RV, right ventricle; SLS, septal longitudinal strain; sPAP, systolic pulmonary artery pressure; TAPSE, tricuspid annulus plane systolic excursion; TDI, tissue Doppler imaging.

^a^Covariates included in multivariable analysis are age and sex.

## Discussion

In this observational study we provide insights into the short-term haemodynamic, biomarker, and echocardiographic effects of intermittent levosimendan infusion in patients with AdHF. We found a non-significant reduction in MAP values at 30 days and an improvement of laboratory data as NT-proBNP and eGFR after 48 h from infusion which lasts for around 30 days. Cardiac parameters showed a rapid improvement in left and right ventricular function at 48 h after the infusion, followed by a gradual decline at 30 days. Improvements of LV and RV function assessed by deformation imaging at 48 h were significantly associated with less probability of clinical outcomes. Patients with AdHF may be treated with extended inotropic support as intermittent infusions of levosimendan, to improve symptoms.^9–11^ Given the long half-life of its pharmacodynamically active metabolite, levosimendan administered intermittently in hospital or in ambulatory setting has prolonged pharmacological effects after initial infusion. However, few studies investigated the changes of cardiac parameters assessed by standard and advanced 2d echocardiography in AdHF patients treated with intermittent infusions of levosimendan.^12–14^ Lunghetti *et al.* analysed 41 patients with AdHF treated with levosimendan. The authors observed an improvement in LV longitudinal function and in LVEF with a reduction of *E*/*e*′ values after treatment with levosimendan.^14^ Oner *et al.*^13^ analysed 61 patients with AdHF treated with levosimendan vs dobutamine. In the levosimendan group, LV systolic and diastolic diameters and left atrial diameter were significantly reduced. Moreover, there was an improvement in LVEF and a reduction of *E*/*e*′ values. Another study evaluated 50 patients with AdHF in which levosimendan was given monthly on a 24-h intravenous protocol for 6 months. At the end of the study, the levosimendan group had a significant increase in LVEF, LV shortening fraction and a decrease in mitral regurgitation.^12^ Lastly, meta-analyses reported an improvement of LVEF,^16^ RV function and decrease of the pulmonary artery pressure.^17^

Our analysis confirms the reported data and extends prior reports by analysing a comprehensive and serial assessment of clinical and cardiac changes after levosimendan infusion. We found a significant improvement of parameters of systolic and diastolic LV function, of the RV function parameters and the RV-arterial coupling. However, our analysis showed how the effect is drastically reduced at 30 days from administration showing the need for infusions at least every 2 weeks.

Less is known on the association between cardiac changes after levosimendan infusion and clinical outcomes. In our population, early ΔLVGLS and ΔRVFWLS were significantly associated with clinical outcomes including CV death and worsening HF. Myocardial strain analysis is less load dependent than LVEF or TAPSE/FAC and is calculated in semi-automatic mode, hence, is less operator dependent. Moreover, myocardial strain takes into account not just the net longitudinal displacement of the basal segments towards the apex but also the underlying twisting motion.^18^ This tool may capture subclinical changes in LV function not detected by standard measures able to identify patients who better respond to medical treatment. Previous data showed that LVGLS was the strongest function parameter correlated with the severity of myocardial fibrosis in AdHF.^19,20^ Similarly, the analysis of RV longitudinal deformation was also found to be related to myocardial fibrosis.^21^ Our observations support the hypothesis that the degree of functional improvement following levosimendan infusion may reflect the extent of underlying myocardial fibrosis, with greater responsiveness potentially identifying patients more likely to undergo favourable cardiac remodelling. Improvement in LVGLS may also reflect enhanced ventriculo–arterial coupling and myocardial efficiency following levosimendan infusion. By combining calcium sensitization with vasodilatory effects, levosimendan reduces afterload and improves myocardial–vascular interaction, potentially allowing a more effective conversion of myocardial contraction into forward flow. This improved mechanical efficiency may contribute to better deformation patterns and, ultimately, to a more favourable clinical outcome. A reduction in left ventricular filling pressures may have led to a temporary improvement in renal perfusion, a potential mechanism that deserves further exploration in longer-term studies.

The results of this study have several potential clinical implications. The early changes in LVGLS and RVFWLS and the association with CV events suggest that deformation imaging can provide early prognostic insights beyond conventional echocardiographic parameters. From this perspective the routine use of speckle-tracking strain analysis may help clinicians to identify responders to levosimendan therapy and personalize treatment strategies.

## Limitations

This study has several limitations. First, all clinical and echocardiographic data were collected by local investigators based on available medical records, without centralized adjudication of laboratory results or echocardiographic parameters by a core laboratory. This may have introduced variability in data acquisition and interpretation. Second, the relatively small sample size may have limited the statistical power to detect more nuanced associations and restricted the ability to perform subgroup analyses. Third, the absence of a control population precludes direct comparisons and limits the generalizability of our findings to broader patient populations. Future prospective studies with larger cohorts, standardized imaging protocols, and controlled designs are warranted to validate these observations.

## Conclusions

In a contemporary cohort of patients with AdHF, intermittent levosimendan infusion was associated with early improvements in NT-proBNP levels as well as left and right ventricular functional parameters at 48 h. However, these effects appeared to attenuate by 30 days. Notably, early improvements in biventricular function were associated with a lower risk of adverse clinical outcomes, suggesting a potential prognostic value. These findings may assist clinicians in identifying patients most likely to derive meaningful clinical benefit from intermittent levosimendan therapy.

## Supplementary Material

xvag017_Supplementary_Data

## Data Availability

No data were generated or analysed for this manuscript.

## Pre-registered clinical trial number

None supplied.
