# Supplementary material for: Clinical and echocardiographic changes after intermittent levosimendan infusion in patients with advanced heart failure
Source: ESC Heart Fail. 2026 Jan 13;13(1):xvag017. doi: 10.1093/eschf/xvag017 (PMC13108296; doi:10.1093/eschf/xvag017)
Supplement: xvag017_Supplementary_Data [file xvag017_supplementary_data.docx]

**Supplementary material**

**Supplementary Table S1: Demographic and clinical characteristics stratified by the occurrence of the composite outcome.**

|  | Non-outcome  (n = 20) | Outcome  (n = 17) | p-value |
| --- | --- | --- | --- |
| **Demographics and clinical characteristics** | | | |
| Age, years | 72.6 + 8.5 | 69.4 + 9.5 | 0.290 |
| Male, n (%) | 12 (60.0) | 10 (58.8) | 0.942 |
| BMI, Kg/m^2^ | 24.9 + 3.8 | 27.0 + 5.8 | 0.212 |
| SBP, mmHg | 112.8 + 12.8 | 110.3 + 15.8 | 0.604 |
| MAP, mmHg | 82.8 + 9.9 | 79.5 + 8.9 | 0.306 |
| Heart rate, bpm | 69.3 + 11.2 | 65.8 + 10.5 | 0.341 |
| Hypertension, n (%) | 8 (40.0) | 9 (52.9) | 0.431 |
| Dyslipidaemia, n (%) | 10 (50.0) | 11 (64.7) | 0.368 |
| Diabetes, n (%) | 7 (35.0) | 13 (76.5) | **0.012** |
| Coronary artery disease, n (%) | 10 (50.0) | 13 (76.5) | 0.098 |
| Prior STEMI, n (%) | 8 (40.0) | 7 (41.2) | 0.942 |
| Atrial fibrillation, n (%) | 13 (65.0) | 10 (58.8) | 0.699 |
| Chronic kidney disease, n (%) | 17 (85.0) | 14 (82.4) | 0.828 |
| Stage III | 13 (65.0) | 10 (58.8) |  |
| Stage IV | 3 (15.0) | 4 (23.5) |  |
| COPD, n (%) | 5 (25.0) | 6 (35.3) | 0.495 |
| Chronic anaemia, n (%) | 3 (15.0) | 3 (17.7) | 0.828 |
| Non-ischaemic cardiomyopathy, n (%) | 6 (30.0) | 4 (23.5) | 0.659 |
| Primary VHD, n (%) | 3 (15.0) | 1 (5.9) | 0.373 |
| ICD, n (%) | 9 (45.0) | 9 (52.9) | 0.630 |
| CRT-D, n (%) | 8 (40.0) | 9 (52.9) | 0.431 |
| MitraClip, n (%) | 1 (5.0) | 2 (11.8) | 0.452 |
| **Medical therapy** | | | |
| ACEi, n (%) | / | 1 (5.9) | 0.272 |
| ARB, n (%) | 1 (5.0) | 1 (5.9) | 0.906 |
| ARNI, n (%) | 13 (65.0) | 7 (41.2) | 0.147 |
| MRA, n (%) | 19 (95.0) | 16 (94.1) | 0.906 |
| β-blockers, n (%) | 18 (90.0) | 16 (94.1) | 0.647 |
| Diuretics, n (%) | / | / | / |
| Ivabradine, n (%) | 1 (5.0) | 2 (11.8) | 0.452 |
| **Laboratory tests** | | | |
| NT-proBNP, ng/L | 2036 (1399 – 4014) | 1490 (993 - 5212) | 0.446 |
| Hs-TnT, ng/L | 18 (13 – 33) | 24 (16 – 38) | 0.514 |
| Creatinine, mg/dL | 1.66 + 0.59 | 2.11 + 1.01 | 0.097 |
| eGFR, mL/min | 45.9 + 21.8 | 37.6 + 19.3 | 0.235 |

Data are presented as n (% on available), as mean (+SD) and as median (IQR). Bold values represent significant p-values.
ACEi, angiotensin converting enzyme inhibitor; ARB, angiotensin receptor blocker; ARNI, angiotensin receptor neprilysin inhibitor; BMI, body mass index; COPD, chronic obstructive pulmonary disease; CRT-D, cardiac resynchronization therapy - defibrillator; eGFR, estimated glomerular filtration rate; hs-TnT, high-sensitivity cardiac troponin T; ICD, implantable cardioverter defibrillator; MAP, mean arterial pressure; MRA, mineralocorticoid receptor antagonist; NT-proBNP, N-terminal pro b-type natriuretic peptide; SBP, systolic blood pressure; SGLT2i, sodium-glucose cotransporter 2 inhibitor; STEMI, ST elevation myocardial infarction; VHD, valvular heart disease.

**Supplementary Table S2: Outcome according to baseline echocardiographic parameters**

|  | Non-outcome  (n = 20) | Outcome  (n = 17) | p-value |
| --- | --- | --- | --- |
| LV-EDV, mL | 187.2 + 17.9 | 203.7 + 17.5 | 0.516 |
| LV-EDVi, mL/m^2^ | 103.0 + 8.9 | 112.2 + 10.1 | 0.500 |
| LV-EDD, mm | 60.0 + 2.1 | 64.7 + 2.3 | 0.135 |
| LVEF, % | 34.8 + 2.8 | 27 + 2.0 | **0.048** |
| Stroke volume, mL | 49.6 + 3.0 | 51.3 + 3.9 | 0.728 |
| LVOT-VTI, cm | 15.7 + 1.1 | 13.8 + 1.1 | 0.234 |
| LV-GLS, % | -8.9 + 0.8 | -6.9 + 0.8 | 0.082 |
| LAV, mL | 114.0 + 8.7 | 107.2 + 9.1 | 0.599 |
| LAVi mL/m^2^ | 63.2 + 4.5 | 58.4 + 4.3 | 0.446 |
| RAV, mL | 87.4 + 16.4 | 72.9 + 8.6 | 0.463 |
| E/e’ | 16.4 + 1.6 | 18.9 + 1.9 | 0.324 |
| Moderate-severe MR, n (%) | 6 (35.3) | 5 (25.0) | 0.495 |
| TAPSE, mm | 17.3 + 0.7 | 16.5 + 0.7 | 0.428 |
| S’wave TDI, cm/s | 9.3 + 0.5 | 8.4 + 0.4 | 0.148 |
| FAC, % | 36.4 + 2.4 | 32.1 + 2.5 | 0.228 |
| sPAP, mmHg | 48.8 + 3.3 | 48.5 + 4.3 | 0.945 |
| TAPSE/sPAP, mm/mmHg | 0.39 + 0.03 | 0.38 + 0.04 | 0.940 |
| RV FWLS, % | -17.5 + 1.6 | -17.9 + 1.6 | 0.875 |
| RV SLS, % | -8.3 + 1.1 | -8.9 + 1.7 | 0.773 |

Data are presented as n (% on available) and as mean (+SD). Bold values represent significant p-values.
EDD, end-diastolic diameter; EDV, end-diastolic volume; EDVi, end-diastolic volume indexed; FAC, fractional area change; FWLS, free wall longitudinal strain; GLS, global longitudinal strain; LAV, left atrium volume; LAVi, left atrium volume indexed; LV, left ventricle; LVEF, left ventricular ejection fraction; LVOT-VTI, left ventricular outflow tract – velocity time integral; MR, mitral regurgitation; RAV, right atrium volume; RV, right ventricle; SLS, septal longitudinal strain; sPAP, systolic pulmonary artery pressure; TAPSE, tricuspid annulus plane systolic excursion; TDI, tissue doppler imaging.

**Supplementary Table S3: Outcome according to echocardiographic parameters variation post- and pre- levosimendan infusion**

|  | Overall population (n = 37) | Non-outcome (n = 20) | Outcome (n = 17) | p-value |
| --- | --- | --- | --- | --- |
| ΔLV-EDV, mL | -7.16 + 15.41 | -3.65 + 8.99 | -11.29 + 20.11 | 0.135 |
| ΔLV-EDV indexed, mL/m^2^ | -3.91 + 8.92 | -2.01 + 5.13 | -6.13 + 11.75 | 0.165 |
| ΔLV-EDD, mm | -0.65 + 1.46 | -0.40 + 0.99 | -0.94 + 1.85 | 0.266 |
| ΔLVEF, % | 1.27 + 2.52 | 0.85 + 2.66 | 1.76 + 2.33 | 0.278 |
| ΔStroke volume, mL | 1.39 + 7.25 | 2.47 + 3.53 | 0.18 + 9.89 | 0.350 |
| ΔLVOT-VTI, cm | 0.83 + 3.51 | 1.49 + 3.08 | 0.09 + 3.92 | 0.237 |
| ΔLV GLS, % | 1.13 + 1.10 | 1.61 + 0.96 | 0.58 + 1.01 | **0.003** |
| ΔLAV, mL | -4.35 + 8.94 | -1.95 + 6.83 | -7.18 + 10.42 | 0.076 |
| ΔLAV indexed, mL/m^2^ | -2.38 + 4.66 | -1.10 + 3.51 | -3.89 + 5.45 | 0.069 |
| ΔRAV, mL | -0.43 + 5.51 | -2.10 + 4.82 | 1.53 + 5.75 | **0.044** |
| ΔE/e’ | -2.26 + 4.89 | -2.21 + 3.72 | -2.32 + 6.10 | 0.946 |
| ΔTAPSE, mm | 0.76 + 1.48 | 0.95 + 1.70 | 0.53 + 1.18 | 0.396 |
| ΔS’wave TDI, cm/s | 0.74 + 1.30 | 0.8 + 1.20 | 0.68 + 1.45 | 0.778 |
| ΔFAC, % | 1.57 + 4.34 | 1.7 + 3.19 | 1.41 + 5.50 | 0.844 |
| ΔsPAP, mmHg | -7.03 + 9.23 | -6.68 + 5.66 | -7.41 + 12.26 | 0.817 |
| ΔTAPSE/sPAP, mm/mmHg | 0.08 + 0.11 | 0.11 + 0.13 | 0.05 + 0.08 | 0.153 |
| ΔRV-FWLS, % | 1.48 + 3.75 | 2.76 + 3.47 | -0.02 + 3.59 | **0.022** |
| ΔRV-SLS, % | 1.07 + 2.72 | 1.45 + 3.37 | 0.48 + 0.92 | 0.337 |

Data are presented as n (% on available) and as mean (+SD). Bold values represent significant p-values.
EDD, end-diastolic diameter; EDV, end-diastolic volume; EDVi, end-diastolic volume indexed; FAC, fractional area change; FWLS, free wall longitudinal strain; GLS, global longitudinal strain; LAV, left atrium volume; LAVi, left atrium volume indexed; LV, left ventricle; LVEF, left ventricular ejection fraction; LVOT-VTI, left ventricular outflow tract – velocity time integral; MR, mitral regurgitation; RAV, right atrium volume; RV, right ventricle; SLS, septal longitudinal strain; sPAP, systolic pulmonary artery pressure; TAPSE, tricuspid annulus plane systolic excursion; TDI, tissue doppler imaging.

**Supplementary Figure S1: change in main cardiac parameters according to outcome**

 

LAVi, left atrial volume indexed; LVEDVi, left ventricular end-diastolic volume indexed; LVEF, left ventricular ejection fraction; LVGLS, left ventricular global longitudinal strain; LVOT-VTI, left ventricular outflow tract – velocity time integral; sPAP, systolic pulmonary arterial pressure
